# Yoga as Potential Therapy for Burnout: Health Technology Assessment Report on Efficacy, Safety, Economic, Social, Ethical, Legal and Organizational Aspects

**DOI:** 10.1007/s11920-024-01516-1

**Published:** 2024-09-13

**Authors:** Marleen Schröter, Holger Cramer, Heidemarie Haller, Stefan Huster, Ulrike Lampert, Martin Schaefer, Gesa Janssen-Schauer, Friedhelm Meier, Anja Neumann, Silke Neusser, Anna K. Koch

**Affiliations:** 1 Charité Competence Center for Traditional and Integrative Medicine (CCCTIM), Charité – Universitätsmedizin Berlin, Corporate Member of Freie Universität Berlin, Humboldt-Universität zu Berlin and Berlin Institute of Health, Augustenburger Platz 1, 13353 Berlin, Germany; 2 Institute of General Practice and Interprofessional Care, University Hospital Tübingen, Tübingen, Germany; 3 Bosch Health Campus, Stuttgart, Germany; 4 Center for Integrative Medicine and Planetary Health, University Hospital Essen, University of Duisburg-Essen, Essen, Germany; 5 Ruhr-University of Bochum, Bochum, Germany; 6 Institute for Quality and Efficiency in Health Care (IQWiG), Cologne, Germany; 7 Department of Psychiatry, Psychotherapy, Psychosomatics and Addiction Medicine, Kliniken Essen-Mitte, Essen, Germany; 8 Department of Psychiatry, Charité Campus Mitte, Charité Universitätsmedizin Berlin, corporate member of Freie Universität Berlin, Humboldt-Universität zu Berlin, and Berlin Institute of Health, Berlin, Germany; 9 Institute for Ethics, Faculty of Protestant Theology, University of Tübingen, Tübingen, Germany; 10 Research Institute for Medicine Management GmbH, Essen, Germany

**Keywords:** Yoga_1_, Burnout_2_, Benefit assessment_3_, Systematic review_4_, Health technology assessment_5_

## Abstract

**Purpose of Review:**

This health technology assessment aimed to systematically assess the efficacy and safety of yoga as therapy for burnout. Economic, ethical, legal, social and organizational aspects were considered as well.

**Recent Findings:**

Yoga as a therapy has been shown to have positive effects on a range of symptoms, including stress, anxiety and depression. Regarding work-related stress and burnout, the effects of yoga have mainly been examined in a preventative context.

**Summary:**

Meta-analyses revealed no effects on burnout severity comparing yoga with passive controls in general. Compared with passive controls, yoga had a positive effect on subjective stress. Compared to active control, yoga had an effect on the burnout subscale depersonalization on individual study level. Yoga may have positive effects on burnout, but the results are mixed. Common definitions and standardized diagnostic tools are necessary to improve research and further assess yoga as therapy for burnout.

**Trial Registration:**

The HTA is registered with PROSPERO, CRD42022299405, on 6th February 2022.

**Supplementary Information:**

The online version contains supplementary material available at 10.1007/s11920-024-01516-1.

## Introduction

Burnout is a globally increasing health issue with negative implications for health and performance [1–4]. The COVID-19 pandemic has exacerbated the situation even more [5, 6]. There are different approaches to prevent and treat burnout, from pharmacotherapy and psychotherapy to stress-reducing methods such as relaxation, mindfulness, and art therapy [7–12]. However, there is no generally recognized, standardized form of therapy or prevention. Another intervention that is receiving increasing attention in the field of stress-related disorders is yoga. The growing body of applied yoga research in recent decades shows that yoga as therapy has positive effects on a range of psychological and physical symptoms, including, stress, anxiety, depression, sleep disorders, quality of life and pain [13–16]. The effect of yoga on anxiety and depressive symptoms has been studied in meta-analyses [13, 17]. Here, positive effects were found less in manifest clinical affective disorders, but mainly in subsyndromal depressive and anxiety symptoms. For depressive disorders, yoga may be superior to pure relaxation and physical exercises [13]. In addition to the antidepressant and health-promoting effects of physical activity in general, as also practised in yoga, the neurophysiological mechanisms of the breathing- and meditation-based elements of yoga possibly also play an important role in stress-associated diseases [18, 19]. In the context of work-related stress and burnout, there are already some diverse approaches to the use of yoga mainly used in a preventive manner, from individual to group-based interventions, integrated in the work context, in combination with other approaches such as art, music or mindfulness and self-compassion elements [20]. However, the effect of yoga as therapy on burnout has not been systematically reviewed yet. The aim of this health technology assessment (HTA) was to systematically review and metaanalyze the efficacy and safety of yoga as a therapy for burnout.

## Methods

This HTA was planned and conducted in accordance with PRISMA (Preferred Reporting Items for Systematic Reviews and Meta-Analyses) [21] guidelines and the recommendations of the Cochrane Collaboration [22]. The methodology is in accordance with the general methods of the Institute for Quality and Efficiency in Healthcare in Germany (IQWIG), version 6.1 [23]. The HTA report without the partial search update from 2023 was published in German on the funder´s website (IQWIG: https://www.iqwig.de/sich-einbringen/themencheck-medizin/berichte/ht21-02.html). The publication of the results on the sfunder´s website was a condition for the funding. The HTA is registered with PROSPERO, CRD42022299405.

### Eligibility Assessment

For the efficacy and safety analyses, randomized controlled trials (RCTs), cluster-randomized trails, and randomized cross-over studies in German or English were included. Studies were included if adults (≥ 18 years) diagnosed with burnout (including diagnosis code Z73.0 ICD-10 "Burnout"), adults with elevated burnout levels on validated burnout scales (e.g. Maslach Burnout Inventory (MBI) [24]) at baseline, a population with mean elevated levels of burnout on validated burnout scales (e.g. MBI) at baseline, or employees who were unable to work due to diagnosed burnout, were studied. Interventions were included if they were explicitly labelled as "yoga" or "yogic". No restrictions were placed on the yoga tradition, length, frequency or duration of the program. Studies investigating yoga as an add-on were also included. Studies comparing yoga to (1) no treatment, (2) treatment as usual or (3) an active control intervention were eligible. Efficacy outcomes were defined as *severity of burnout, remission, subjective stress, depressive symptoms, self-efficacy, health-related quality of life, mortality* and *adverse events*. Economic, ethical, legal, social and organizational aspects were considered as well. Titles and abstracts identified during the database and hand searches were screened by two reviewers (AKK, HH) independently, with potentially eligible articles read in full by two reviewers (AKK, HH) to determine whether they met the eligibility criteria. Disagreements were discussed with a third reviewer (MS or HC) until consensus was reached. If necessary, additional information was obtained from the study authors.

### Search Strategies

#### Efficacy and Safety

Ovid MEDLINE(R), Embase (via Ovid), Cochrane Central Register of Controlled Trials, PsycINFO, ClinicalTrials.gov, and the World Health Organization international clinical trials registry platform were searched from inception through November 29th, 2021. A search update was executed in Embase until May 9th, 2023 by AKK and MS (See supplement Tables 1 to 6 for search strategies).

#### Economic, Ethical, Legal, Social and Organizational Aspects

A systematic literature search for comparative health economic studies was performed in MEDLINE, EMBASE, and HTA database. Calculation of intervention costs was based on literature and expert interviews as well as German prices. To review the ethical aspects, reference was made to the identified efficacy studies, an orienting search was performed using the relevant databases (ETHMED, MEDLINE) and information from laws, regulations or directives as well as interest-dependent sources of information, for example websites of interest representatives were evaluated.

To review the social and organizational aspects, orienting searches were performed in MEDLINE and on websites of relevant institutions and associations (Robert-Koch institute, Professional Association of Yoga Teachers in Germany e. V., Yoga Vidya). To review legal aspects, an orienting search was performed using Juris, the leading online portal for legal and practical knowledge in Germany.

### Data Extraction and Management

#### Efficacy and Safety

Data on participants (age, gender, diagnosis), methods (randomization, allocation concealment), interventions (yoga style, frequency, and duration), control interventions (type, frequency, duration), outcomes (outcome measures, questionnaires, assessment time points), and results were independently extracted by two reviewers (AKK and MS) using an a priori data extraction form. Discrepancies were discussed with a third reviewer (HC) until consensus was reached. If necessary, study authors were contacted for additional information.

#### Risk of Bias of Individual Studies

The risk of selection bias, performance bias, detection bias, attrition bias, reporting bias, and other source of bias regarding efficacy studies were independently assessed by two reviewers (AKK and MS) using the IQWIG risk of bias appraisal [23]. Each domain was assessed as either, ‘yes’, ‘no’ or ‘unclear’. Discrepancies were discussed with a third reviewer (HC) until consensus was reached.

#### Economic, Ethical, Legal, Social and Organizational Aspects

The health economic evaluation considered intervention costs and cost-effectiveness.

The ethical evaluation considered statements on ethical aspects and arguments of yoga interventions [25, 26]. The evaluation of legal aspects was based on Brönneke 2016 [27]. Social and sociocultural aspects addressed the reciprocal interactions between examination or treatment methods and the social environment [28]. The evaluation of organizational aspects was based on Perleth 2014 [29].

### Statistical Analysis

Regarding efficacy and safety, if three or more studies were available, IQWIG methodology was used to determine whether analyses should be performed using the Knapp-Hartung method or the DerSimonian-Laird method [23]. Factors possibly causing this heterogeneity were also investigated. Because of the small number of studies, neither subgroup nor sensitivity analyses nor assessment of publication bias were performed. Statistical heterogeneity between the study effects was analyzed by the test of heterogeneity with a *p*-value of ≤ .05 indicating significant heterogeneity. The magnitude of heterogeneity was categorized by the I^2^ with I^2^ > 25%, I^2^ > 50%, and I^2^ > 75% representing moderate, substantial, and considerable heterogeneity, respectively [30]. All analyses were performed using the Statistical Package for Social Sciences software (IBM SPSS Statistics for Windows, release 29.0; IBM Corporation, Armonk, NY).

## Results

### Efficacy and Safety

**Fig. 1:**
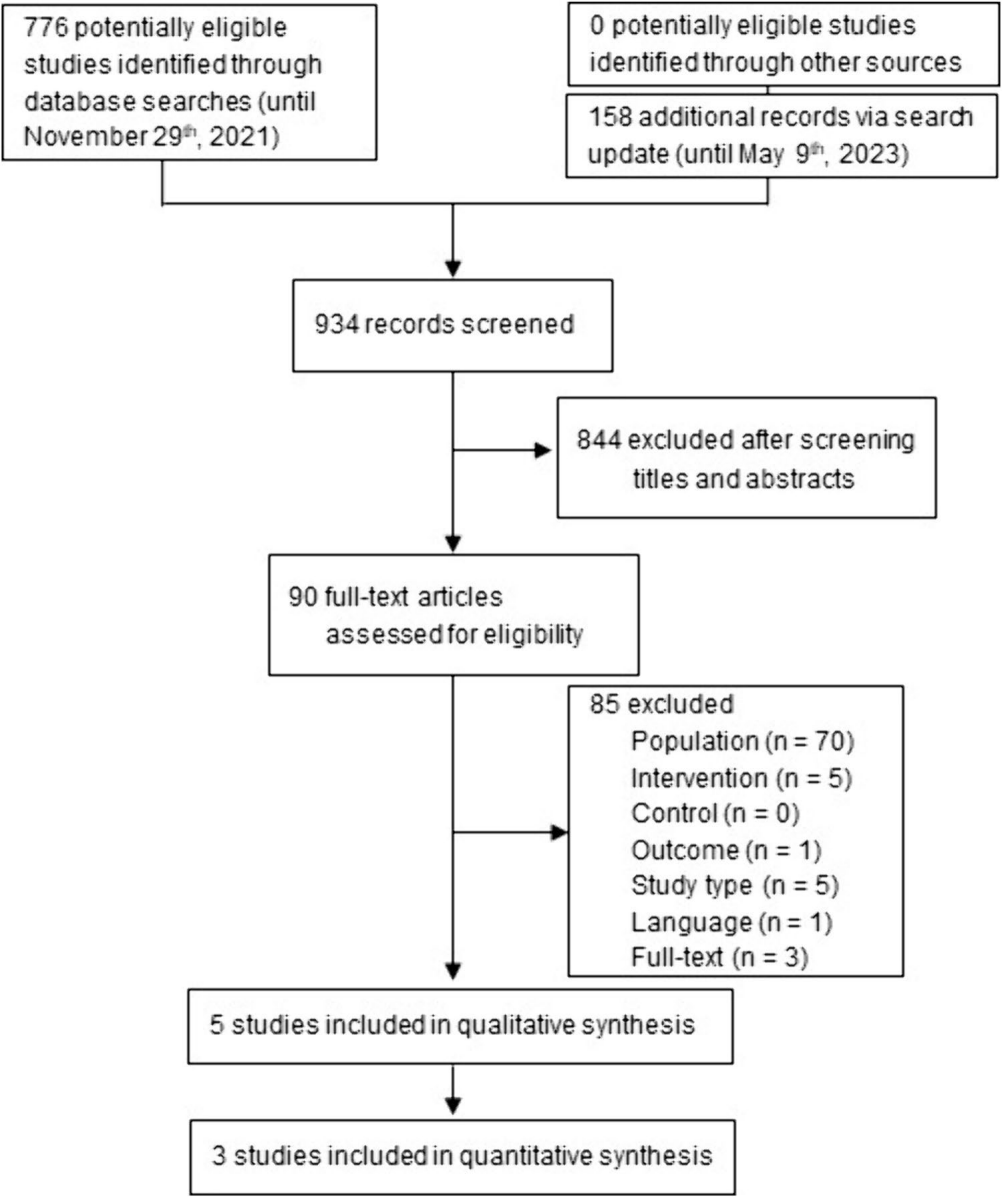
Flowchart

The systematic literature search for primary studies concerning efficacy and safety yielded 776 results that were initially screened, the search update via Embase yielded additional 158 records (Fig. 1). Of these, 934 were excluded based on information in the title or abstract. Overall, 90 studies were screened for eligibility by reading the full text (supplement). The final study pool consisted of five studies [31–35].

The characteristics of the five included RCTs are presented in detail in Table 1. Two studies compared Yoga with an active control intervention (mindfulness-based cognitive therapy, cognitive behavioral therapy and group-fitness classes) [31, 32] and three compared Yoga with a passive control intervention (no treatment, treatment as usual, waiting-list) [33–35]. The Yoga interventions differed in terms of intervention length (3–20 weeks) and content (traditional yoga [31], trauma-informed Hatha Yoga [32], medical yoga + usual care [33], yoga + mindfulness [34] and a structured yoga program [35]). Studies were conducted in Sweden [31], Australia [32], Germany [33], USA [34] and India [35]. All studies included participants with mean elevated burnout baseline-scores or on sick leave due to burnout. All studies provided data directly after the end of the intervention. Details on efficacy outcomes and the specific scales used in the respective studies can be found in supplement Tables 7 and 8.

**Table 1:** Study characteristics

**Study**	**Study design**	**N**	**Intervention**	**Control**	**Place, time of implementation**	**Study length**	**Outcomes**
**Active control group**							
Grensman et al. (2018) [31]	RCT	94	Traditional-Yoga	(1) Mindfulness-based cognitive therapy (2) Cognitive behavioral therapy	Karolinska Institute, Stockholm, Sweden 09/2007 to 11/2009	20 weeks	Primary: Health-related quality of life Secondary: Adverse events
Taylor et al. (2020) [32]	RCT Pilot	21	Trauma-informed Hatha Yoga	Group fitness classes (interval and/or boxing fit training)	Royal Prince Alfred Hospital, Camperdown, Australia 11/2018 to 7/2019	8 weeks	Primary: Burnout Secondary: Adverse events
**Passive control group**							
Köhn et al. (2013) [33]	RCT	39	Medical Yoga + Standard-therapy	Standard therapy	Center for Primary Health Care, Germany 03/2011 to 06/2011	12 weeks	Primary: Subjective stress Secondary: Burnout severity, depressive symptoms, adverse events
Ancona and Mendelson (2014) [34]	Cluster RCT	52 (only 43 reported)	Yoga + Mindfulness	No intervention	Public schools in Baltimore City, USA Study period not specified	3 weeks	Stress, burnout
Mandal et al. (2021) [35]	RCT	113	Structured Yoga Program	Waiting list	Centre for Community Medicine, AIIMS, Ansarinagar, Delhi, India 05/05/2018–25/06/2018	12 weeks	Primary: Subjective stress Secondary: Occupational quality of life (compassion exhaustion, burnout, secondary traumatic stress^a^), adverse events Drop-out due to adverse events

^a^Only the burnout subscale was relevant and was used for the efficacy assessment

#### Yoga vs. Active Control

No meta-analyses were possible for the comparison yoga vs. active control. At the individual study level with respect to burnout measures*,* significant group differences in favor of Yoga were evident for depersonalization (DP) measured by MBI-DP, (*p* = .05) [32]. No significant differences regarding the burnout-subscales emotional exhaustion (EE) measured by MBI-EE, personal accomplishment (PA) measured by MBI-PA, and burnout measured by the Professional Quality of Life scale (PROQOL) were evident [32]. Regarding health-related quality of life, group differences were not statistically significant [31].

#### Yoga vs. Passive Control

Three studies were included for the meta-analysis on burnout severity [33–35]. The meta-analysis with Knapp-Hartung correction revealed no significant effects (Hedges´g = -0.41, *p* = .18, 95% confidence interval (CI) [-1,29; 0.46]). Heterogeneity was not substantial (Q = 2.5, *p* = .29). Three studies were included for the meta-analysis on subjective stress [33–35]. Heterogeneity was substantial (Q = 10, *p* = .01), a pooled effect is therefore not calculated (CI between -11.08 to 9.00, Hedges´g of single studies between -0.23 and -1.66, thus above irrelevance threshold of 0.2). Accordingly, the effect size was not in a certainly irrelevant range and a qualitative evidence synthesis was conducted, revealing a difference with moderate effects. Regarding depressive symptoms, no significant group differences were evident on the individual study level [33].

#### Adverse Events

Four studies assessed adverse events but data was insufficiently reported in the studies and not suitable for further assessment [31–33, 35].

### Risk of Bias of Individual Studies

Random sequence generation and allocation concealment was assessed as unclear or inadequate in one study [34]. Due to the nature of included studies involving interventions that were obvious to both, participants and personnel, neither of them could be blinded (i.e., high risk in all studies). Selective reporting was assessed as unclear in three studies [31, 33, 34] and possible risk of other sources of bias was assessed as high in one study [34]. Table 2 displays bias assessments for all studies.

**Table 2:** Risk of bias on the individual study level

**Study**	**Adequate generation of the rando-mization sequence**	**Concealment of group allocation**	**Blinding**		**Results-independent reporting**	**Absence of other aspects**	**Potential for bias across endpoints**
			**Patients**	**Treating persons**			
**Active control group**							
Grensman et al. (2018) [31]	yes	yes	no	no	unclear	no	high
Taylor et al. (2020) [32]	yes	yes	no	no	yes	no	high
**Passive control group**							
Köhn et al. (2013) [33]	yes	yes	no	no	unclear	no	high
Ancona and Mendelson (2014) [34]	unclear	no	no	no	unclear	yes	high
Mandal et al. (2021) [35]	yes	yes	no	no	yes	no	high

### Assessment of the Scope of Unpublished Data

334 ongoing studies were identified, 36 were potentially suitable in terms of content, of which 35 were still in the process of conduction at the time of the search. For one study, results were already available, reported in the study register. This study did not meet the inclusion criteria, however.

### Economic, Social, Ethical, Legal and Organizational Aspects

The systematic literature search for studies concerning health economy yielded 27 results (supplement Fig. 1). Ten full texts were screened, none fulfilled eligibility criteria. Therefore the cost-effectiveness could not be evaluated. The average cost of a yoga course in Germany is 148 to 226 € for a 12-week course of 60 and 90 min, respectively, per treatment case. Regarding social and organizational aspects, Yoga as a hobby experiences an increasing interest and demand, the offer especially in urban regions increases accordingly [36, 37]. More women practice yoga compared to men [37], the percentage of yoga practitioners is highest among middle-aged singles, middle aged persons living together in partnerships without children, and young seniors. There are also more yoga practitioners among people with higher education, civil servants and employees [37]. Of those currently practicing yoga, 86% reported a perceived change as a result of their practice. The most frequently cited changes were a sense of balance/calmness/relaxation, and feeling "better" [37]. From the patient's perspective, yoga is seen as a way to positively influence their own health [37]. The main reasons mentioned for starting a yoga practice were relaxation, prevention/health promotion, and treatment of a health problem. The main reasons for continuing the yoga practice were prevention/health promotion, spirituality and relaxation [38]. Barriers to yoga practice included work and family commitments, incompatibility with lifestyle, lack of family support, and lack of yoga options [39]. Fear of injury and a lacking sense of self-efficacy may also be obstacles for practicing yoga [40]. Concerning the ethical aspects, the secondary literature refers to the religious roots of yoga [41, 42]. Even though yoga is applied in a medical context largely independent of its religious origins [43], patients with a certain religious education can be expected to have reservations about yoga as a therapeutic intervention [44]. Ethically, a heightened ideological sensitivity of yoga teachers towards patients is thus required. From a legal perspective, it is problematic that yoga courses are basically not regulated. There are numerous different yoga styles and the term "yoga teacher" is not protected. The quality of yoga needs to be made more transparent [45, 46]. Yoga associations provide certified yoga trainings to some extent, but in part the acceptance of certain trainings is simply based on a good reputation of the provider in the sector [46]. However, there is an opportunity for yoga schools to be certified by the International Yoga Alliance^®^. This certification offers a possibility for quality assurance and standardization of yoga training. The establishment of appropriate structures and procedures for obtaining a certification as yoga therapist is necessary. The international association of yoga therapists (IAYT) [47], for example, advocates a certification for individual yoga therapists that is internationally recognized and respected, meets certain standards, and is intended to ensure the quality of the work of yoga therapists. So far, this training is primarily offered in the USA. The training to become a yoga therapist is usually financed by the trainees themselves.

## Discussion

This HTA includes 5 RCTs from five countries that examined the efficacy of yoga as a therapy for burnout [31–35]. For yoga compared with passive controls, short-term effects were shown on depersonalization, a subcomponent of burnout, but not on burnout severity in general. Compared with active controls, yoga showed a positive effect on subjective stress. Adverse events were insufficiently reported. When safety issues were reported, yoga did not result in more adverse events than usual care and comparable adverse events to active controls. Our findings suggest that yoga as a therapy may have beneficial effects on burnout, but results are inconsistent.

An overview of systematic reviews and meta-analyses on burnout reduction in physicians and nurses included 22 reviews [48]. These 22 reviews evaluated both behavioral interventions that focus on the individual (including yoga), and structural interventions (e.g., workplace-related interventions such as job rotations). The authors concluded that a combination of different interventions was most effective. However, the overview also included studies that focused on the preventive rather than therapeutic aspects of yoga for burnout. The conclusions of this overview can therefore only be linked to those of the present HTA to a very limited extent. A systematic review and network meta-analysis on relaxation techniques for occupational stress included fifteen randomized controlled trials, seven of them on yoga [49]. Here, yoga was identified as the most effective method for stress reduction. Another systematic review evaluated studies on whether yoga can decrease stress and burnout in nurses. Seven clinical trials were included, and the conclusion tended to be positive for yoga [20]. However, the results of these reviews are hardly applicable to the question of the present HTA: No consistent distinction was made between prevention and therapy of burnout, other stress-associated indications are considered in addition to burnout, and yoga interventions are not evaluated exclusively. This limited comparison with the existing overviews and reviews on the topic of yoga on burnout, clearly shows the deficits in this research field: The reviews and also the primary studies themselves rarely distinguish between prevention and therapy.

This is mainly due to the complexity of diagnosing burnout; the concept of burnout faces conceptual and methodological difficulties, such as its multidimensionality, absence of a uniform definition and standardized measures [3, 7, 50]. There is no generally recognised definition of burnout. A position paper of the German Society for Psychiatry, Psychotherapy and Neurology (DGPPN) dealt with the topic of burnout [51]. According to the DGPPN, the definition most frequently used in research and practice is based on the symptom triad (1) emotional exhaustion, (2) depersonalisation and (3) reduced work performance. The most widely used instrument to assess burnout is the MBI, which is consistent with these three subscales [24]. Within the Diagnostic and Statistical Manual of Mental Disorders, Fifth Edition (DSM-5) [52] and the ICD-10-F [53] no diagnosis for burnout exists, often the Z-diagnosis “Problems related to life management difficulty” is applied. Due to the lack of definition and the absence of an F-diagnosis in ICD-10, precise prevalence data are hardly possible. Symptoms are often closely related to depressive and anxiety disorders so that a clear distinction is often not possible [53].

Yoga as a hobby is experiencing an increasing demand and is practiced by an increasing number of people [36, 37]. From the patient's perspective, it is seen as a way to positively influence their own health [38]. However, it is only partially subsidized by health insurance companies in Germany as a preventive measure; as a therapy for burnout, the costs are not covered by health insurance companies. From a legal point of view, it is problematic that yoga courses are practically not regulated. There are numerous different yoga variations and the title "yoga teacher" is not protected. Ethically, a heightened ideological sensitivity of yoga teachers towards patients is further required. There is a need here to ensure that the quality of yoga offers is made more transparent, a certified yoga training for patients with burnout is desirable.

In yoga studies, heterogeneity of yoga interventions such as yoga style, length and frequency and insufficient reporting of delivered yoga programs further limit the interpretation of results, making it impossible to distinguish effective characteristics of the intervention for example. Rigorous methodology and reporting in yoga studies is warranted to evaluate the effects of different components of yoga on health outcomes.

### Strengths and Limitations

The present HTA specifically addresses the therapeutic efficacy of yoga for burnout, rather than preventive aspects. Therefore, studies were included which, according to a priori defined inclusion criteria, exclusively studied populations with clinically diagnosed burnout or elevated burnout levels measured by validated scales. Further, the study of possible therapy methods requires a comprehensive perspective. This was done by addressing economic, ethical, legal, social, and organizational aspects, in addition to an efficacy evaluation. Also, conclusions are not restricted due to unpublished data as this was considered as well.

However, results should be interpreted with caution. Reasons include the low number of studies investigating the effects of yoga in this study population, the overall unclear study quality, and the often missing systematic assessment and reporting of adverse events. It was often not possible to pool data due to the small number of studies. Also sensitivity and subgroup analysis were not possible due to the small number of studies. As no standardized cut-off scores for burnout exist, inclusion criteria were based on the cut-offs reported by Doulougeri et al. [54] and do not represent generally accepted cut-offs. The study by Ancona and Mendelson [34] does not indicate whether a vote by an ethics committee was available. Currently, a vote by an ethics committee is not a binding requirement for open trials [55, 56]. However, since this is also research on and with humans, this is ethically problematic. In the future, it can be expected that votes of ethics committees will also be an indispensable prerequisite for independent studies. Since informed consent from all healthy participants was available in this study, it seemed ethically justified to include the study in the benefit assessment.

### Implications for Future Research

A social, ethical and organizational challenge is the diagnosis of burnout due to the unspecific variety of symptoms, unclear definition and lack of standardized measurement tools. A standardized diagnostic of burnout is needed for future studies. A clear distinction should be made between preventive and therapeutic approaches.

## Conclusion

Yoga as a therapy may have positive effects on burnout symptoms and subjective stress but results are inconsistent. Common definitions, classifications and standardized diagnostic tools of burnout are necessary to improve scientific research on this topic and further assess yoga as a therapy method for burnout.

## Supplementary Information

Below is the link to the electronic supplementary material.

Supplementary file1 (DOCX 83 KB)

## Data Availability

Data can be obtained from the corresponding author on request.
