# Supplementary material for: Yoga as Potential Therapy for Burnout: Health Technology Assessment Report on Efficacy, Safety, Economic, Social, Ethical, Legal and Organizational Aspects
Source: Curr Psychiatry Rep. 2024 Sep 13;27(12):723–32. doi: 10.1007/s11920-024-01516-1 (PMC12592297; doi:10.1007/s11920-024-01516-1)
Supplement: Supplementary file 1 — Supplementary file1 (DOCX 83 KB) [file 11920_2024_1516_MOESM1_ESM.docx]

Supplementary Material

Yoga as Potential Therapy for Burnout: Health Technology Assessment Report on Efficacy, Safety, Economic, Social, Ethical, Legal and Organizational Aspects

Current Psychiatry Reports

**Marleen Schröter, Holger Cramer, Heidemarie Haller, Stefan Huster, Ulrike Lampert, Martin Schaefer, Gesa Janssen-Schauer, Friedhelm Meier, Anja Neumann, Silke Neusser, Anna K. Koch^*^**

*** Correspondence:** Dr. rer. nat. Anna Katharina Koch^1^: [anna-katharina.koch@charite.de](mailto:anna-katharina.koch@charite.de)

^1^ Charité Competence Center for Traditional and Integrative Medicine (CCCTIM), Charité – Universitätsmedizin Berlin, Corporate Member of Freie Universität Berlin, Humboldt-Universität zu Berlin and Berlin Institute of Health, Berlin, Germany.

Supplementary Table 1. Search Strategy Embase

| # | Searches |
| --- | --- |
| 1 | exp stress/ |
| 2 | (stress* or burnout*).ti,ab. |
| 3 | or/1-2 |
| 4 | exp yoga/ |
| 5 | yoga*.ti,ab. |
| 6 | or/4-5 |
| 7 | 3 and 6 |
| 8 | (random* or double-blind*).tw. |
| 9 | placebo*.mp. |
| 10 | or/8-9 |
| 11 | 7 and 10 |
| 12 | 11 not medline.cr. |
| 13 | 12 not (exp animal/ not exp human/) |
| 14 | 13 not (Conference Abstract or Conference Review or Editorial).pt. |
| 15 | 14 not ((afrikaans or albanian or arabic or armenian or azerbaijani or basque or belorussian or bosnian or bulgarian or catalan or chinese or croatian or czech or danish or dutch or english or esperanto or estonian or finnish or french or gallegan or georgian or german or greek or hebrew or hindi or hungarian or icelandic or indonesian or irish gaelic or italian or japanese or korean or latvian or lithuanian or macedonian or malay or norwegian or persian or polish or polyglot or portuguese or pushto or romanian or russian or scottish gaelic or serbian or slovak or slovene or spanish or swedish or thai or turkish or ukrainian or urdu or uzbek or vietnamese) not (english or german)).lg. |

Supplementary Table 2. Search Strategy Cochrane Library

| # | Searches |
| --- | --- |
| #1 | [mh "Stress, Psychological"] |
| #2 | (stress* or burnout*):ti,ab |
| #3 | #1 or #2 |
| #4 | [mh ^"Yoga"] |
| #5 | yoga*:ti,ab |
| #6 | #4 OR #5 |
| #7 | #3 AND #6 |
| #8 | #7 not (*clinicaltrial*gov* or *who*trialsearch* or *clinicaltrialsregister*eu* or *anzctr*org*au* or *trialregister*nl* or *irct*ir* or *isrctn* or *controlled*trials*com* or *drks*de*):so |
| #9 | #8 not ((language next (afr or ara or aze or bos or bul or car or cat or chi or cze or dan or dut or es or est or fin or fre or gre or heb or hrv or hun or ice or ira or ita or jpn or ko or kor or lit or nor or peo or per or pol or por or pt or rom or rum or rus or slo or slv or spa or srp or swe or tha or tur or ukr or urd or uzb)) not (language near/2 (en or eng or english or ger or german or mul or unknown))) in Trials |

Supplementary Table 3. Search Strategy PsychInfo

| # | Searches |
| --- | --- |
| 1 | exp stress/ |
| 2 | (stress* or burnout*).ti,ab. |
| 3 | or/1-2 |
| 4 | Yoga/ |
| 5 | yoga*.ti,ab. |
| 6 | or/4-5 |
| 7 | 3 and 6 |
| 8 | (double-blind or randomized or randomly assigned).tw. |
| 9 | 7 and 8 |
| 10 | 9 not ((albanian or arabic or bulgarian or catalan or chinese or croatian or czech or danish or dutch or english or estonian or farsi iranian or finnish or french or georgian or german or greek or hebrew or hindi or hungarian or italian or japanese or korean or lithuanian or malaysian or nonenglish or norwegian or polish or portuguese or romanian or russian or serbian or serbo croatian or slovak or slovene or spanish or swedish or turkish or ukrainian or urdu) not (english or german)).lg. |

Supplementary Table 4. Search Strategy ClinicalTrials.gov

| Search Strategy |
| --- |
| AREA[ConditionSearch] ( stress OR burnout ) AND AREA[InterventionSearch] yoga |

Supplementary Table 5. Search Strategy World Health Organization International Clinical Trials Registry Platform

| Search Strategy |
| --- |
| (stress OR burnout) AND yoga [without synonyms] |

Supplementary Table 6 Search strategy Ovid MEDLINE(R).

| # | Searches |
| --- | --- |
| 1 | exp Stress, Psychological/ |
| 2 | (stress* or burnout*).ti,ab. |
| 3 | or/1-2 |
| 4 | Yoga/ |
| 5 | yoga*.ti,ab. |
| 6 | or/4-5 |
| 7 | randomized controlled trial.pt. |
| 8 | controlled clinical trial.pt. |
| 9 | (randomized or placebo or randomly or trial or groups).ab. |
| 10 | drug therapy.fs. |
| 11 | or/7-10 |
| 12 | 11 not (exp animals/ not humans.sh.) |
| 13 | and/3,6,12 |
| 14 | (animals/ not humans/) or comment/ or editorial/ or exp review/ or meta analysis/ or consensus/ or exp guideline/ |
| 15 | hi.fs. or case report.mp. |
| 16 | or/14-15 |
| 17 | 13 not 16 |
| 18 | 17 and (english or german or multilingual or undetermined).lg. |

**Supplementary Table 7.** Outcomes efficacy

| Outcome category | Value pre-study | | | | | Value post-study | | | Mean difference pre-post | | | Intervention vs. Control | | | Information |
| --- | --- | --- | --- | --- | --- | --- | --- | --- | --- | --- | --- | --- | --- | --- | --- |
|  | Mean | | SD | | n / N | Mean | SD | n / N | Mean | | SD | difference | [95 %-CI] | p-value |  |
| Severity of burnout | | | | | | | | | | | | | | | |
| Active Control | | | | | | | | | | | | | | | |
| Taylor et al. (2020) |  | |  | |  |  |  |  |  | |  |  |  |  | Maslach Burnout Inventory (MBI), Human Services Survey for Medical Personnel; emotional exhaustion (EE), Depersona-lisation (DP), personal accomplishment (PA); Professional Quality of Life Scale (PROQOL) - Burnout |
| Trauma-informed Hatha Yoga | 27 (MBI-EE)  14 (MBI-DP)  33 (MBI-PA)  27 (PROQOL) | | 11 (MBI-EE  )  6 (MBI-DP)  6 (MBI-PA)  4 (PROQOL) | | 10/11 | 25 (MBI-EE)  10 (MBI- DP)  36 (MBI-PA)  25 (PROQOL) | 10 (MBI-EE)  6 (MBI-DP)  6 (MBI-PA)  5 (PROQOL) | 10/11 | -2 (MBI-  EE)  -4 (MBI-DP)  3 (MBI-PA)  -2 (PROQOL) | | n.r. | -0,69 (MBI-EE) ^a^  -1,99 (MBI-DP) ^a^  0,64 (MBI-PA) ^a^  -0,73 (PROQOL) ^a^ | n.r. | 0,49 (MBI-EE) ^a^  0,05 (MBI-DP) ^a^  0,52 (MBI-PA) ^a^  0,47 (PROQOL) ^a^ |  |
| Fitness-Group | 21 (MBI-EE)  10 (MBI-DP)  32 (MBI-PA)  27 (PROQOL) | | 9 (MBI-EE)  7 (MBI-DP)  8 (MBI-PA)  4 (PROQOL) | | 8/8 | 21 (MBI-EE)  11 (MBI-DP)  36 (MBI-PA)  25 (PROQOL) | 12 (MBI-EE)  7 (MBI-DP)  7 (MBI-PA)  6 (PROQOL) | 8/8 | 0 (MBI-EE)  1 (MBI-DP)  4 (MBI-PA)  -2 (PROQOL) | | n.r. |  |  |  |  |
| Passive Control | | | | | | | | | | | | | | | |
| Köhn et al. (2013) |  | |  | |  |  |  |  |  | |  |  |  |  | Shirom-Melamed Burnout Question-naire |
| Medical Yoga | 4,4 | | 1,2 | | 18 / 20 | 3,2 | 0,6 | 18 / 20 | -1,1 | | n.r. | 0,1 | -0,4 bis 0,7 | 0,412 |  |
| Usual care | 4,7 | | 0,8 | | 19 / 19 | 3,7 | 0,6 | 19 / 19 | -1,0 | | n.r. |  |  |  |  |
| Ancona & Mendelson (2014) | | | | | | | | | | | | | | | |
| Yoga- and mindfulness intervention | 31,48 | | 10,04 | | 21/n.r. | 29,81 | 8,50 | 21/n.r. | -1,67 | | 4,02 | -1,39 | - | 0,17 | The Maslach Burnout Inventory-Educators Survey- emotional exhaustion |
| No treatment | 30,05 | | 12,51 | | 22/n.r. | 30,68 | 10,81 | 22/n.r. | 0,63 | | 6,60 |  |  |  |  |
| Mandal et al. (2021) |  | |  | |  |  |  |  |  | |  |  |  |  | Professional Quality of Life Scale - Burnoutsubskala |
| Structured yoga program | 58,7 | | 6,5 | | 58 / 58 | 54,6 | 5,3 | 19 / 58 | 5,3 | | 6,6 | -1,9 | -0,8 bis 0,4 | 0,142 |  |
| Waiting list | 56,9 | | 4,9 | | 52 / 55 | 56,5 | 4,1 | 32 / 55 | 1,3 | | 6,1 |  |  |  |  |
| Subjective Stress | | | | | | | | | | | | | | | |
| Passive Control | | | | | | | | | | | | | | | |
| Köhn et al. (2013) |  |  | |  | |  |  |  |  |  | |  |  |  | Perceived Stress Scale |
| Medical Yoga | 36,3 | 10,2 | | 18 / 20 | | 18,7 | 6,7 | 18 / 20 | -17,7 | n.r. | | 15,5 | 8,1 bis 23,0 | <0,001 |  |
| Usual care | 34,2 | 10,7 | | 19 / 19 | | 32,1 | 8,9 | 19 / 19 | -2,2 | n.r. | |  |  |  |  |
| Ancona & Mendelson (2014) |  | |  | |  |  |  |  |  | |  |  |  |  | Teacher Stress Inventory |
| Yoga- and mindfulness intervention | 2,85 | | 0,74 | | 21/n.r. | 2,56 | 0,63 | 21/n.r. | -0,29 | | 0,42 | -1,8 |  | 0,08 |  |
| No treatment | 2,75 | | 0,61 | | 22/n.r. | 2,71 | 0,64 | 22/n.r. | -0,4 | | 0,50 |  |  |  |  |
| **Mandal et al. (2021)** |  | |  | |  |  |  |  |  | |  |  |  |  | Perceived Stress Scale |
| Structured yoga program | 20,7 | | 5,9 | | 58 / 58 | 15,5 | 5,4 | 19 / 58 | 6,3 | | 8,6 | -1,3 | -1,9 bis -0,7 | 0,0003 |  |
| Waiting list | 19,8 | | 4,7 | | 52 / 55 | 20,7 | 2,8 | 30 / 55 | -0,9 | | 4,5 |  |  |  |  |
| **Depressiveness** | | | | | | | | | | | | | | | |
| Passive Control | | | | | | | | | | | | | | | |
| Köhn et al. (2013) |  | |  | |  |  |  |  |  | |  |  |  |  | Hospital Anxiety and Depression Scale |
| Medical Yoga | 8,2 | | 5,1 | | 18 / 20 | 4,1 | 3,8 | 18 / 20 | -4,1 | | n.r. | 2,1 | -0,2 bis 4,6 | 0,123 |  |
| Usual care | 8,5 | | 3,9 | | 19 / 19 | 6,6 | 4,3 | 19 / 19 | -1,9 | | n.r. |  |  |  |  |
| **Health-related quality of life** | | | | | | | | | | | | | | | |
| Active Control | | | | | | | | | | | | | | | |
| Grensman et al. (2018)^a^ |  | |  | |  |  |  |  |  | |  |  |  |  | Swedish health-related  quality of life survey, 1.0 |
| Traditional Yoga (TY) | 86 (PF)  33 (SPF)  50 (P)  33 (RLP)  11 (RLE)  40 (PA)  33 (NA)  29 (CF)  36 (SL)  44 (GH)  38 (SFF)  58 (SFP)  45 (SF) | | 81-90 (PF)  33-33 (SPF)  38-62 (P)  18-48 (RLP)  3-26 (RLE)  25-56 (PA)  22-45 (NA)  14-44 (CF)  24-47 (SL)  37-51 (GH)  15-60 (SFF)  42-75 (SFP)  30-60 (SF) | | 26/32 | n.r. | n.r. |  |  | |  | n.r. | n.r. | TY vs. CBT: ,28 (PF)  ,82 (SPF)  ,39 (P)  ,76 (RLP)  ,25 (RLE)  ,94 (PA)  ,09 (NA)  1 (CF)  ,40 (SL)  ,41 (GH)  ,14 (SFF)  ,60 (SFP)  ,39 (SF)  TY vs, MBCT:  ,70 (PF)  ,71 (SPF)  ,66 (P)  ,75 (RLP)  ,95 (RLE)  ,70 (PA)  ,65 (NA)  ,35 (CF)  ,66 (SL)  ,69 (GH)  ,46 (SFF)  ,87 (SFP)  ,91 (SF) |  |
| Mindfulness-based cognitive therapy (MBCT) | 81 (PF)  33 (SPF)  33 (P)  53 (RLP)  33 (RLE)  42 (PA)  21 (NA)  13 (CF)  46 (SL)  53 (GH)  55 (SFF)  67 (SFP)  40 (SF) | | 74-88 (PF)  16-50 (SPF)  16-50 (P)  42-63 (RLP)  22-44 (RLE)  31-52 (PA)  17-25 (NA)  2-23 (CF)  37-55 (SL)  34-72 (GH)  35-75 (SFF)  49-84 (SFP)  33-47 (SF) | | 27/31 | n.r. | n.r. |  |  | |  |  |  |  |  |
| Cognitive behavior therapy (CBT) | 90 (PF)  33 (SPF)  62 (P)  33 (RLP)  33 (RLE)  33 (PA)  29 (NA)  25 (CF)  43 (SL)  50 (GH)  40 (SFF)  58 (SFP)  48 (SF) | | 86-95 (PF)  16-50 (SPF)  49-67 (P)  27-39 (RLP)  22-44 (RLE)  17-50 (PA)  21-38 (NA)  17-33 (CF)  34-52 (SL)  36-64 (GH)  23-56 (SFF)  47-70 (SFP)  29-66 (SF) | | 27/31 | n.r. | n.r. |  |  | |  |  |  |  |  |
| CI: confidence interval; n: Number of patients analyzed; N: number of patients randomized; SD: Standard deviation; n.r.: not reported; ^a^Z-adjusted for age and gender  PF = Physical functioning; SPF = Satisfaction with physical functioning; P = Pain; RLP = Role limitations due to physical health; RLE = Role limitations due to emotional health; PA = Positive affect; NA = Negative affect; CF = Cognitive functioning; SL = Sleep; GH = General health; SFF = Satisfaction with family functioning; SFP = Satisfaction with partner functioning; SF = Sexual functioning; TY = traditional yoga; CBT = Cognitive Behavior Therapy; MBCT = mindfulness-based cognitive therapy  ^a^ Data refers to medians, minimum - maximum | | | | | | | | | | | | | | | |

**Supplementary Table 8.** Details on the specific scales used in the respective studies to assess burnout severity, subjective stress, depressiveness, and health-related quality of life

| **Instrument** | **Study** | | | | | **Information** |
| --- | --- | --- | --- | --- | --- | --- |
|  | Taylor et al. (2020) | Köhn et al. (2013) | Ancona & Mendelson (2014) | Mandal et al. (2021) | Grensman et al. (2018) | Value range and interpretation |
| **Severity of burnout** | | | | | | |
| Maslach Burnout Inventory, Human Services Survey for Medical Personnel (MBI-HSS (MP)); Emotional Exhaustion (EE) | x |  |  |  |  | 0-54 (higher scores indicating a higher severity of burnout) |
| MBI-HSS (MP)- Depersonalisation (DP) | x |  |  |  |  | 0-30 (higher scores indicating a higher severity of burnout) |
| MBI-HSS (MP) personal accomplishment (PA) | x |  |  |  |  | 0-48 (lower scores indicating a higher severity of burnout) |
| Professional Quality of Life Scale – Burnout scale | x |  |  | x |  | 10-50 (higher scores indicating a higher severity of burnout) |
| Shirom-Melamed Burnout Questionnaire |  | x |  |  |  | 22-154 (higher scores indicating a higher severity of burnout) |
| The Maslach Burnout Inventory-Educators Survey-(MBI-ES) emotional exhaustion (EE) |  |  | x |  |  | 0-54 (higher scores indicating a higher severity of burnout) |
| **Subjective Stress** | | | | | | |
| Perceived Stress Scale (PSS) |  | x |  | x |  | 0-40 (higher scores indicating a higher subjective stress) |
| Teacher Stress Inventory (TSI) |  |  | x |  |  | 49-245 (higher scores indicating a higher subjective stress) |
| **Depressiveness** | | | | | | |
| Hospital Anxiety and Depression Scale (HADS) |  | x |  |  |  | 0-21 (higher scores indicating a higher depressiveness) |
| **health-related quality of life** | | | | | | |
| Swedish health-related  quality of life survey (SWED-QUAL), 1.0 |  |  |  |  | x | 0-100 (higher scores indicating a higher health-realted quality of life) |

# Supplementary Data

**2.1 Excluded full-texts and reasons for exclusion: efficacy and safety**

**2.1.1 Population**

1. Abinaya P, Indupriya S. A study on occupational stress of assistant professors in selected private universities - Chennai. Indian Journal of Public Health Research and Development 2019; 10(4): 197-201. https://dx.doi.org/10.5958/0976-5506.2019.00689.2.

2. Alexander GK, Rollins K, Walker D et al. Yoga for Self-Care and Burnout Prevention Among Nurses. Workplace Health &Safety 2015; 63(10): 462-470; quiz 471. https://dx.doi.org/10.1177/2165079915596102.

3. Axen I, Follin G. Medical yoga in the workplace setting-perceived stress and work ability-a feasibility study. Complementary therapies in medicine 2017; 30: 61‐66. https://dx.doi.org/10.1016/j.ctim.2016.12.001.

4. Benejam CJB. A comparison of the effects of thermal biofeedback training and yoga practice on the stress levels of college students. Dissertation Abstracts International: Section B: The Sciences and Engineering 2013; 74(6-B(E)): No Pagination Specified.

5. Bhandari RB. Yogic intervention for coping with distress. Journal of clinical and diagnostic research 2017; 11(12): Oc06‐oc11. https://dx.doi.org/10.7860/jcdr/2017/29332.10944.

6. Biman S, Maharana S, Metri KG et al. Effects of yoga on stress, fatigue, musculoskeletal pain, and the quality of life among employees of diamond industry: A new approach in employee wellness. Work 2021; 70(2): 521-529. https://dx.doi.org/10.3233/wor-213589.

7. Bock BC, Fava JL, Gaskins R et al. Yoga as a complementary treatment for smoking cessation in women. Journal of Women's Health 2012; 21(2): 240-248. https://dx.doi.org/10.1089/jwh.2011.2963.

8. Bressington D, Mui J, Yu C et al. Feasibility of a group-based laughter yoga intervention as an adjunctive treatment for residual symptoms of depression, anxiety and stress in people with depression. Journal of Affective Disorders 2019; 248: 42-51. https://dx.doi.org/10.1016/j.jad.2019.01.030.

9. Casey LJ, Van Rooy KM, Sutherland SJ et al. Improved Self-Acceptance, Quality of Life, and Stress Level from Participation in a Worksite Yoga Foundations Program: A Pilot Study. International Journal of Yoga Therapy 2018; 28(1): 15-21. https://dx.doi.org/10.17761/2018-00013r2.

10. Cheema BS, Houridis A, Busch L et al. Effect of an office worksite-based yoga program on heart rate variability: outcomes of a randomized controlled trial. BMC Complementary &Alternative Medicine 2013; 13: 82. https://dx.doi.org/10.1186/1472-6882-13-82.

11. Chu IH, Wu WL, Lin IM et al. Effects of Yoga on Heart Rate Variability and Depressive Symptoms in Women: A Randomized Controlled Trial. Journal of Alternative &Complementary Medicine 2017; 23(4): 310-316. https://dx.doi.org/10.1089/acm.2016.0135.

12. Collins LA. Stress management and yoga. Dissertation abstracts international 1984; 45(1‐a): 0116.

13. Danucalov MAD, Kozasa EH, Ribas KT et al. A Yoga and compassion meditation program reduces stress in familial caregivers of alzheimer's disease patients. Evidence-based Complementary and Alternative Medicine 2013; 2013: 513149. https://dx.doi.org/10.1155/2013/513149.

14. Daukantaite D, Tellhed U, Maddux RE et al. Five-week yin yoga-based interventions decreased plasma adrenomedullin and increased psychological health in stressed adults: A randomized controlled trial. PLoS ONE [Electronic Resource] 2018; 13(7): e0200518. https://dx.doi.org/10.1371/journal.pone.0200518.

15. de Manincor M, Bensoussan A, Smith CA et al. Individualized Yoga for Reducing Depression and Anxiety, and Improving Well-Being: A Randomized Controlled Trial. Depression &Anxiety 2016; 33(9): 816-828. https://dx.doi.org/10.1002/da.22502.

16. Descilo T, Vedamurtachar A, Gerbarg PL et al. Effects of a yoga breath intervention alone and in combination with an exposure therapy for post-traumatic stress disorder and depression in survivors of the 2004 South-East Asia tsunami. Acta Psychiatrica Scandinavica 2010; 121(4): 289-300. https://dx.doi.org/10.1111/j.1600-0447.2009.01466.x.

17. Duchemin AM, Steinberg BA, Marks DR et al. A small randomized pilot study of a workplace mindfulness-based intervention for surgical intensive care unit personnel: effects on salivary alpha-amylase levels. Journal of Occupational &Environmental Medicine 2015; 57(4): 393-399. https://dx.doi.org/10.1097/jom.0000000000000371.

18. Eda N, Ito H, Akama T. Beneficial Effects of Yoga Stretching on Salivary Stress Hormones and Parasympathetic Nerve Activity. Journal of Sports Science &Medicine 2020; 19(4): 695-702.

19. Falsafi N. A Randomized Controlled Trial of Mindfulness Versus Yoga: Effects on Depression and/or Anxiety in College Students. Journal of the American Psychiatric Nurses Association 2016; 22(6): 483-497. https://dx.doi.org/10.1177/1078390316663307.

20. Fang R, Li X. A regular yoga intervention for staff nurse sleep quality and work stress: a randomised controlled trial. Journal of Clinical Nursing 2015; 24(23-24): 3374-3379. https://dx.doi.org/10.1111/jocn.12983.

21. Gandhi S, Palled VK, Sahu M et al. Effectiveness of caregivers'yoga module on psychological distress and mental well-being among caregivers of patients admitted to neurological rehabilitation wards of a tertiary care institute, bengaluru, karnataka, india. Journal of Neurosciences in Rural Practice 2019; 10(4): 657-665. https://dx.doi.org/10.1055/s-0039-3399613.

22. Gomathi B. Effectiveness of selected yoga practices on the level of stress among student nurses in selected nursing educational institution, Dehradun. European Journal of Molecular and Clinical Medicine 2020; 7(8): 1450-1456.

23. Granath J, Ingvarsson S, von Thiele U et al. Stress management: a randomized study of cognitive behavioural therapy and yoga. Cognitive Behaviour Therapy 2006; 35(1): 3-10. https://dx.doi.org/10.1080/16506070500401292.

24. Hamza A, Jagannathan A, Hegde S et al. Development and Testing of an Audio-Visual Self-Help Yoga Manual for Indian Caregivers of Persons with Schizophrenia Living in the Community: A Single-Blind Randomized Controlled Trial. International Journal of Yoga 2020; 13(1): 62-69. https://dx.doi.org/10.4103/ijoy.IJOY_70_18.

25. Harkess KN, Delfabbro P, Mortimer J et al. Brief report on the psychophysiological effects of a yoga intervention for chronic stress: preliminary findings. Journal of psychophysiology 2017; 31(1): 38‐48. https://dx.doi.org/10.1027/0269-8803/a000169.

26. Harris AR, Jennings PA, Katz DA et al. Promoting stress management and wellbeing in educators: Feasibility and efficacy of a school-based yoga and mindfulness intervention. Mindfulness 2016; 7(1): 143-154. https://dx.doi.org/10.1007/s12671-015-0451-2.

27. Hartfiel N, Burton C, Rycroft-Malone J et al. Yoga for reducing perceived stress and back pain at work. Occupational Medicine (Oxford) 2012; 62(8): 606-612. https://dx.doi.org/10.1093/occmed/kqs168.

28. Hartfiel N, Havenhand J, Khalsa SB et al. The effectiveness of yoga for the improvement of well-being and resilience to stress in the workplace. Scandinavian Journal of Work, Environment &Health 2011; 37(1): 70-76. https://dx.doi.org/10.5271/sjweh.2916.

29. Hewett ZL, Pumpa KL, Smith CA et al. Effect of a 16-week Bikram yoga program on perceived stress, self-efficacy and health-related quality of life in stressed and sedentary adults: A randomised controlled trial. Journal of Science &Medicine in Sport 2018; 21(4): 352-357. https://dx.doi.org/10.1016/j.jsams.2017.08.006.

30. Hilcove K, Marceau C, Thekdi P et al. Holistic Nursing in Practice: Mindfulness-Based Yoga as an Intervention to Manage Stress and Burnout. Journal of Holistic Nursing 2021; 39(1): 29-42. https://dx.doi.org/10.1177/0898010120921587.

31. Huang FJ, Chien DK, Chung UL. Effects of Hatha yoga on stress in middle-aged women. Journal of Nursing Research 2013; 21(1): 59-66. https://dx.doi.org/10.1097/jnr.0b013e3182829d6d.

32. Hylander F, Johansson M, Daukantaite D et al. Yin yoga and mindfulness: a five week randomized controlled study evaluating the effects of the YOMI program on stress and worry. Anxiety, Stress, &Coping 2017; 30(4): 365-378. https://dx.doi.org/10.1080/10615806.2017.1301189.

33. Javnbakht M, Hejazi Kenari R, Ghasemi M. Effects of yoga on depression and anxiety of women. Complementary Therapies in Clinical Practice 2009; 15(2): 102-104. https://dx.doi.org/10.1016/j.ctcp.2009.01.003.

34. Kanderis Lane CL, Gurenlian JR, Freudenthal J et al. A 15-Minute Yoga Intervention to Reduce Entry-Level Dental Hygiene Student Stress. Journal of Dental Hygiene 2021; 95(2): 63-70.

35. Khalsa SB, Shorter SM, Cope S et al. Yoga ameliorates performance anxiety and mood disturbance in young professional musicians. Applied Psychophysiology &Biofeedback 2009; 34(4): 279-289. https://dx.doi.org/10.1007/s10484-009-9103-4.

36. Klatt M, Norre C, Reader B et al. Mindfulness in motion: A mindfulness-based intervention to reduce stress and enhance quality of sleep in Scandinavian employees. Mindfulness 2017; 8(2): 481-488. https://dx.doi.org/10.1007/s12671-016-0621-x.

37. Klatt M, Steinberg B, Duchemin AM. Mindfulness in Motion (MIM): An Onsite Mindfulness Based Intervention (MBI) for Chronically High Stress Work Environments to Increase Resiliency and Work Engagement. Journal of Visualized Experiments 2015; (101): e52359. https://dx.doi.org/10.3791/52359.

38. Lavretsky H, Epel ES, Siddarth P et al. A pilot study of yogic meditation for family dementia caregivers with depressive symptoms: effects on mental health, cognition, and telomerase activity. International Journal of Geriatric Psychiatry 2013; 28(1): 57-65. https://dx.doi.org/10.1002/gps.3790.

39. Lee H. Yoga improves perceived stress and psychological outcomes in distressed women. Focus on alternative and complementary therapies 2013; 18(4): 217‐218. https://dx.doi.org/10.1111/fct.12055.

40. Lin SL, Huang CY, Shiu SP et al. Effects of Yoga on Stress, Stress Adaption, and Heart Rate Variability Among Mental Health Professionals--A Randomized Controlled Trial. Worldviews on Evidence-Based Nursing 2015; 12(4): 236-245. https://dx.doi.org/10.1111/wvn.12097.

41. Loewenthal J, Dyer NL, Lipsyc-Sharf M et al. Evaluation of a Yoga-Based Mind-Body Intervention for Resident Physicians: A Randomized Clinical Trial. Global Advances in Health &Medicine 2021; 10: 21649561211001038. https://dx.doi.org/10.1177/21649561211001038.

42. Maddux RE, Daukantaite D, Tellhed U. The effects of yoga on stress and psychological health among employees: an 8- and 16-week intervention study. Anxiety, Stress, &Coping 2018; 31(2): 121-134. https://dx.doi.org/10.1080/10615806.2017.1405261.

43. Maglia M, Auditore R, Pipitone S et al. Combining group psychotherapy and yoga exercises improves quality of life in mental health professionals: a controlled randomized clinical trial. Mental Illness 2019; 11(2): 1-7. https://dx.doi.org/10.1108/mij-10-2019-0007.

44. Manocha R, Black D, Sarris J et al. A randomized, controlled trial of meditation for work stress, anxiety and depressed mood in full-time workers. Evidence-Based Complementary &Alternative Medicine: eCAM 2011; 2011: 960583. https://dx.doi.org/10.1155/2011/960583.

45. Mathad MD, Pradhan B, Sasidharan RK. Effect of Yoga on Psychological Functioning of Nursing Students: A Randomized Wait List Control Trial. Journal of Clinical and Diagnostic Research JCDR 2017; 11(5): KC01-KC05. https://dx.doi.org/10.7860/jcdr/2017/26517.9833.

46. Michalsen A, Jeitler M, Brunnhuber S et al. Iyengar yoga for distressed women: a 3-armed randomized controlled trial. Evidence-Based Complementary &Alternative Medicine: eCAM 2012; 2012: 408727. https://dx.doi.org/10.1155/2012/408727.

47. Miyoshi Y. Restorative yoga for occupational stress among Japanese female nurses working night shift: Randomized crossover trial. Journal of Occupational Health 2019; 61(6): 508-516. https://dx.doi.org/10.1002/1348-9585.12080.

48. O'Toole MS, Mennin DS, Applebaum A et al. A randomized controlled trial of emotion regulation therapy for psychologically distressed caregivers of cancer patients. JNCI Cancer Spectrum 2020; 4(1): pkz074. https://dx.doi.org/10.1093/jncics/pkz074.

49. Phansikar M, Mullen SP. Cognitive and psychosocial effects of an acute sun salutation intervention among adults with stress. Mental Health and Physical Activity 2022; 22: 100431. https://dx.doi.org/10.1016/j.mhpa.2021.100431.

50. Rao M, Metri KG, Raghuram N et al. Effects of Mind Sound Resonance Technique (Yogic Relaxation) on Psychological States, Sleep Quality, and Cognitive Functions in Female Teachers: A Randomized, Controlled Trial. Advances in Mind-Body Medicine 2017; 31(1): 4-9.

51. Riley KE, Park CL, Wilson A et al. Improving physical and mental health in frontline mental health care providers: Yoga-based stress management versus cognitive behavioral stress management. Journal of Workplace Behavioral Health 2017; 32(1): 26-48. https://dx.doi.org/10.1080/15555240.2016.1261254.

52. Shankarapillai RNMR. The effect of yoga in stress reduction for dental students performing their first periodontal surgery: a randomized controlled study. International journal of yoga 2012; 5(1): 48‐51.

53. Sharma R, Gupta N, Bijlani RL. Effect of yoga based lifestyle intervention on subjective well-being. Indian Journal of Physiology &Pharmacology 2008; 52(2): 123-131.

54. Smith C, Hancock H, Blake-Mortimer J et al. A randomised comparative trial of yoga and relaxation to reduce stress and anxiety. Complementary Therapies in Medicine 2007; 15(2): 77-83. https://dx.doi.org/10.1016/j.ctim.2006.05.001.

55. Smith JA, Greer T, Sheets T et al. Is there more to yoga than exercise? Alternative Therapies in Health &Medicine 2011; 17(3): 22-29.

56. Telles S, Gupta RK, Bhardwaj AK et al. Increased Mental Well-Being and Reduced State Anxiety in Teachers After Participation in a Residential Yoga Program. Medical Science Monitor Basic Research 2018; 24: 105-112. https://dx.doi.org/10.12659/msmbr.909200.

57. Trent NL, Borden S, Miraglia M et al. Improvements in Psychological and Occupational Well-Being in a Pragmatic Controlled Trial of a Yoga-Based Program for Professionals. Journal of Alternative &Complementary Medicine 2019; 25(6): 593-605. https://dx.doi.org/10.1089/acm.2018.0526.

58. Tsang HW, Cheung WM, Chan AH et al. A pilot evaluation on a stress management programme using a combined approach of cognitive behavioural therapy (CBT) and complementary and alternative medicine (CAM) for elementary school teachers. Stress &Health 2015; 31(1): 35-43. https://dx.doi.org/10.1002/smi.2522.

59. Ullas K, Maharana S, Metri KG et al. Impact of Yoga on Mental Health and Sleep Quality Among Mothers of Children With Intellectual Disability. Alternative Therapies in Health &Medicine 2021; 27(S1): 128-132.

60. Valley MA. Feasibility of a mindfulness-based stress reduction intervention on health care safety. Dissertation Abstracts International: Section B: The Sciences and Engineering 2017; 78(3-B(E)): No Pagination Specified.

61. van Aalst J, Jennen L, Demyttenaere K et al. Twelve-Week Yoga vs. Aerobic Cycling Initiation in Sedentary Healthy Subjects: A Behavioral and Multiparametric Interventional PET/MR Study. Frontiers in Psychiatry 2021; 12: 739356. https://dx.doi.org/10.3389/fpsyt.2021.739356.

62. Van Puymbroeck M, Payne LL, Hsieh PC. A phase I feasibility study of yoga on the physical health and coping of informal caregivers. Evidence-based Complementary and Alternative Medicine 2007; 4(4): 519-529. https://dx.doi.org/10.1093/ecam/nem075.

63. Varambally S, Vidyendaran S, Sajjanar M et al. Yoga-based intervention for caregivers of outpatients with psychosis: A randomized controlled pilot study. Asian Journal of Psychiatry 2013; 6(2): 141-145. https://dx.doi.org/10.1016/j.ajp.2012.09.017.

64. Wadhen V, Cartwright T. Feasibility and outcome of an online streamed yoga intervention on stress and wellbeing of people working from home during COVID-19. Work 2021; 69(2): 331-349. https://dx.doi.org/10.3233/wor-205325.

65. Waechter R, Stahl G, Rabie S et al. Mitigating medical student stress and anxiety: Should schools mandate participation in wellness intervention programs? Medical Teacher 2021; 43(8): 945-955. https://dx.doi.org/10.1080/0142159x.2021.1902966.

66. West J, Otte C, Geher K et al. Effects of Hatha yoga and African dance on perceived stress, affect, and salivary cortisol. Annals of Behavioral Medicine 2004; 28(2): 114-118. https://dx.doi.org/10.1207/s15324796abm2802_6.

67. Wolever RQ, Bobinet KJ, McCabe K et al. Effective and viable mind-body stress reduction in the workplace: a randomized controlled trial. Journal of Occupational Health Psychology 2012; 17(2): 246-258. https://dx.doi.org/10.1037/a0027278.

68. Fischer, J., et al. Stress Reduction by Yoga versus Mindfulness Training in Adults Suffering from Distress: A Three-Armed Randomized Controlled Trial including Qualitative Interviews (RELAX Study). Journal of Clinical Medicine 2022; 11(19).

69. Phansikar, M. and S. Mullen. Cognitive and psychosocial effects of an acute sun salutation intervention among adults with stress. Mental Health and Physical Activity 2022; 22(no pagination).

70. Rajashree, R., et al. Research article An interprofessional collaborative approach to alleviate workplace stress among medical college faculty during COVID pandemic. 2023 Biomedicine 43(1): 450-455.

**2.1.2 Intervention**

1. Asuero AM, Queralto JM, Pujol-Ribera E et al. Effectiveness of a mindfulness education program in primary health care professionals: a pragmatic controlled trial. Journal of Continuing Education in the Health Professions 2014; 34(1): 4-12. https://dx.doi.org/10.1002/chp.21211.

2. Dike IC, Onyishi CN, Adimora DE et al. Yoga complemented cognitive behavioral therapy on job burnout among teachers of children with autism spectrum disorders. Medicine 2021; 100(22): e25801. https://dx.doi.org/10.1097/md.0000000000025801.

3. Ruiz-Iniguez R, Carralero Montero A, Burgos-Julian FA et al. Interactions between Personality and Types of Mindfulness Practice in Reducing Burnout in Mental Health Professionals. International Journal of Environmental Research &Public Health [Electronic Resource] 2021; 18(13). https://dx.doi.org/10.3390/ijerph18136721.

4. Steinberg BA, Klatt M, Duchemin AM. Feasibility of a Mindfulness-Based Intervention for Surgical Intensive Care Unit Personnel. American Journal of Critical Care 2016; 26(1): 10-18. https://dx.doi.org/10.4037/ajcc2017444.

5. Wimmer L, von Stockhausen L, Bellingrath S. Improving emotion regulation and mood in teacher trainees: Effectiveness of two mindfulness trainings. Brain and Behavior 2019; 9(9): e01390. https://dx.doi.org/10.1002/brb3.1390.

**2.1.3 Outcome**

1. Taylor J, Stratton E, McLean L et al. How junior doctors perceive personalised yoga and group exercise in the management of occupational and traumatic stressors. Postgraduate Medical Journal 2021. https://dx.doi.org/10.1136/postgradmedj-2020-139191.

**2.1.4 Study Type**

1. Alvarez E, Sutton A, Barton B et al. Evaluating a group-based Yoga of Stress Resilience programme: a pragmatic before-after interventional study protocol. BMJ Open 2020; 10(3): e035862. https://dx.doi.org/10.1136/bmjopen-2019-035862.

2. Bock BC, Morrow KM, Becker BM et al. Yoga as a complementary treatment for smoking cessation: rationale, study design and participant characteristics of the Quitting-in-Balance study. BMC Complementary &Alternative Medicine 2010; 10: 14. https://dx.doi.org/10.1186/1472-6882-10-14.

3. Braun SE, Deeb G, Carrico C et al. Brief Yoga Intervention for Dental and Dental Hygiene Students: A Feasibility and Acceptability Study. Journal of Evidence-based Integrative Medicine 2019; 24: 2515690X19855303. https://dx.doi.org/10.1177/2515690x19855303.

4. Dyer NL, Borden S, Dusek JA et al. A Pragmatic Controlled Trial of a Brief Yoga and Mindfulness-Based Program for Psychological and Occupational Health in Education Professionals. Complementary Therapies in Medicine 2020; 52: 102470. https://dx.doi.org/10.1016/j.ctim.2020.102470.

5. Taylor, J., et al. "How junior doctors perceive personalised yoga and group exercise in the management of occupational and traumatic stressors." Postgraduate Medical Journal 2021; 98(1161).

**2.1.5 Language**

1. Cho HS. The Effects of Yoga Exercise on Stress and Health status in Clinical Nurses. Korean j rehabil nurs 2004; 7(1): 15‐23.

**1.1.6 Full-text**

1. Effectiveness of mindfulness-based cognitive therapy and conscious group yoga on depression, anxiety, and stress of female caregivers of elderlies with Alzheimer. Annals of tropical medicine and public health 2018; 2(Special ue): 104‐115.

2. Boccia A. Reducing individual stress in the workplace through yoga and the strength deployment inventory: an experimental study. Diss abstr int humanit soc sci 2015; 76(5‐a(e)): No‐Specified.

3. Chhugani KJ, Metri K, Babu N et al. Effects of Integrated Yoga Intervention on Psychopathologies and Sleep Quality Among Professional Caregivers of Older Adults With Alzheimer's Disease: A Controlled Pilot Study. Advances in Mind-Body Medicine 2018; 32(3): 18-22.

**3 Supplementary Figure**

Supplementary Figure 1. Flow-Chart Economic Evaluation.

0 excluded duplicates

0 relevant publications

10 excluded for study type

27 potentially eligible studies identified through bibliographic databases

27 records screened

17 excluded after scanning

titles and abstracts

10 full-text articles

assessed for eligibility
